# Synthetic 18F-FDG PET Image Generation Using a Combination of Biomathematical Modeling and Machine Learning

**DOI:** 10.3390/cancers14112786

**Published:** 2022-06-03

**Authors:** Mohammad Amin Abazari, Madjid Soltani, Farshad Moradi Kashkooli, Kaamran Raahemifar

**Affiliations:** 1 Department of Mechanical Engineering, K. N. Toosi University of Technology, Tehran 19967-15433, Iran; m.amin.abazari@gmail.com (M.A.A.); farshad.moradi@email.kntu.ac.ir (F.M.K.); 2 Faculty of Science, School of Optometry and Vision Science, University of Waterloo, Waterloo, ON N2L 3G1, Canada; kraahemi@gmail.com; 3 Advanced Bioengineering Initiative Center, Multidisciplinary International Complex, K. N. Toosi Univesity of Technology, Tehran 14176-14411, Iran; 4 Center for Biotechnology and Bioengineering (CBB), University of Waterloo, Waterloo, ON N2L 3G1, Canada; 5 Department of Electrical and Computer Engineering, Faculty of Engineering, University of Waterloo, Waterloo, ON N2L 3G1, Canada; 6 Data Science and Artificial Intelligence Program, College of Information Sciences and Technology (IST), Penn State University, State College, PA 16801, USA; 7 Department of Chemical Engineering, University of Waterloo, 200 University Avenue West, Waterloo, ON N2L 3G1, Canada

**Keywords:** synthetic medical image generation, medical image augmentation, deep convolutional generative adversarial networks (DCGANs), [18F]-fluorodeoxyglucose positron emission tomography (18F-FDG PET) imaging, multi-scale computational cancer modeling, tumor-induced angiogenesis, kinetic modeling of diagnostic agents, spatiotemporal modeling

## Abstract

**Simple Summary:**

Training computer-assisted algorithms on medical images, particularly 18F-fluorodeoxyglucose (18F-FDG) positron emission tomography (PET) due to its excellent diagnostic accuracy, is difficult, considering small/fragmented samples or privacy concerns. In computer-vision, deep learning-based models, unlike the conventional data augmentation methods, are highly sought after for creating massive medical samples. For this reason, we developed a multi-scale computational framework to generate synthetic 18F-FDG PET images similar to the real ones in different stages of solid tumor growth and angiogenesis. The framework is developed based on the bio-physiological phenomena of FDG radiotracer uptake in solid tumors using a biomathematical model and a generative adversarial network (GAN)-based architecture. The non-invasive augmented 18F-FDG PET images can be used in clinical practice without the need to manage the patient data. In addition, our spatiotemporal mathematical model can calculate the distribution of various radiopharmaceuticals in different tumor-associated vasculatures.

**Abstract:**

No previous works have attempted to combine generative adversarial network (GAN) architectures and the biomathematical modeling of positron emission tomography (PET) radiotracer uptake in tumors to generate extra training samples. Here, we developed a novel computational model to produce synthetic 18F-fluorodeoxyglucose (18F-FDG) PET images of solid tumors in different stages of progression and angiogenesis. First, a comprehensive biomathematical model is employed for creating tumor-induced angiogenesis, intravascular and extravascular fluid flow, as well as modeling of the transport phenomena and reaction processes of 18F-FDG in a tumor microenvironment. Then, a deep convolutional GAN (DCGAN) model is employed for producing synthetic PET images using 170 input images of 18F-FDG uptake in each of 10 different tumor microvascular networks. The interstitial fluid parameters and spatiotemporal distribution of 18F-FDG uptake in tumor and healthy tissues have been compared against previously published numerical and experimental studies, indicating the accuracy of the model. The structural similarity index measure (SSIM) and peak signal-to-noise ratio (PSNR) of the generated PET sample and the experimental one are 0.72 and 28.53, respectively. Our results demonstrate that a combination of biomathematical modeling and GAN-based augmentation models provides a robust framework for the non-invasive and accurate generation of synthetic PET images of solid tumors in different stages.

## 1. Introduction

The use of artificial intelligence (AI) for the processing and analysis of medical images based on machine learning and deep learning techniques has extensively played a critical role in healthcare over recent years [1,2,3]. It is widely known that larger image datasets lead to better training of deep learning algorithms [4]. However, developing AI-assisted automated models for computer-vision-related tasks such as medical image classification, object detection, and image segmentation is often challenged by a limited training dataset. A lack of a sufficient amount of data generally results in overfitting in which the deep leering models fail to generalize well to additional testing data or predict future observations reliably [4]. In addition, the process of medical image annotation can be costly in terms of time, resources, and effort. It also becomes more difficult for precise labeling in order to identify different stages of tumor growth and angiogenesis. If medical samples are expected to be made public in medical research, obtaining appropriate patient consent for the patients’ protection and interest is necessary [5,6]. Therefore, the medical images available online to the public are limited, and their size and quality are low in most cases [5,6]. Collecting medical images for training an accurate deep learning algorithm is a complex, time-consuming, and costly process that usually requires sufficient funding, obtaining appropriate privacy consent, and the collaboration of technical researchers, radiologists, and clinicians.

Data augmentation is a very powerful solution to the problem of limited data [5,6]. Routine augmentation techniques produce highly correlated image training data by mapping the points of an image to a different position or manipulating the intensity values of the image [6]. Basic data augmentation techniques include several operations, such as geometric transformations, cropping, occlusion, noise injection, and filtering intensity operations data [5,6]. However, the effectiveness of these techniques on medical image analysis with complex imaging textures is not as useful as the conventional image datasets because the medical image patterns might be changed by applying basic geometric and deformable augmentation techniques such as translation and rotation. Synthetic data generation is another type of data augmentation approach that is able to programmatically learn the representations of images, produce realistic images to build the model’s generalizability, and decrease overfitting during the training process [7,8]. Such synthetic generated datasets are extremely useful for medical image analysis. In addition, since they are produced artificially using deep learning models, there are no patient data management concerns.

Among different deep learning architectures used for data augmentation, generative adversarial networks (GANs) [9] are now at the center of public attention because of their great image generation performance. The GAN has two deep architecture functions, generator and discriminator, and it is trained in an adversarial fashion in which the generator produces the fake samples, and the discriminator iteratively trains to distinguish between fake and real samples. Several GAN-based models have been developed for the different application tasks of image segmentation, detection, classification, registration, super-resolution, and denoising [7,8]. Nie et al. [10] proposed an adversarial strategy to train a fully convolutional network, aiming to generate synthetic pelvic computed tomography (CT) images given input magnetic resonance (MR) images. Emami et al. [11] used a method to generate synthetic brain CT datasets using T1-weighted post-Gadolinium MR imaging datasets as the input through a GAN model. Kazuhiro et al. [12] generated synthetic human brain MR images by applying a GAN model, and then quantified the quality of generated images by a visual assessment conducted by five radiologists. Ben-Cohen et al. [13] developed a model to produce synthetic positron emission tomography (PET) images from given input CT images by using a fully convolutional network with a conditional GAN (cGAN). Peng et al. [14] trained a cGAN and a cycle-consistency GAN (cycleGAN) to generate synthetic CT images from MR images of nasopharyngeal carcinoma. Yoo et al. [15] examined the quality of three different deep learning networks named GAN, CycGAN, and reference-guided GAN (RgGAN) for generating synthetic CT images of prostate cancer from T2-weighted MR images.

Some studies have used GAN-based data augmentation models to solve the problem posed by the small sample size or develop multi-classification by generating more synthetic medical images [16,17,18,19,20,21,22,23]. Liu et al. [16] presented a combined approach based on a GAN model and a deep neural network to generate synthetic samples of hepatocellular carcinoma. Han et al. [17] used a two-step GAN-based network to produce and improve MR images of the brain without/with tumors exclusively. Yu et al. [18] used a novel image generation architecture called multiple-channels-multiple-landmarks (MCML), which is based on GANs, to generate color fundus samples from a combination of vessel tree, optic disc, and optic cup. Sun et al. [19] proposed a novel brain MR image synthesis model, i.e., abnormal-to-normal translation GAN (ANT-GAN), to produce a normal-looking medical image according to its abnormal-looking counterpart through non-paired training samples. Islam and Zhang [20] proposed an unsupervised deep learning model to generate synthetic brain PET samples for three different stages of Alzheimer’s disease via a deep convolutional GAN (DCGAN). Similar work has been done by Sajjad et al. [21], who developed a DCGAN model to produce artificial PET images for all three stages of Alzheimer’s disease and then also offered a quantitative validation approach for the GAN to measure the quality of the augmented datasets. Andreini et al. [22] trained a progressively growing GAN (PGGAN) to generate the semantic label-maps and then used an image-to-image translation model to synthesize realistic high-quality retinal images from the generated blood vessel structure. In summary, most of the aforementioned studies have focused on producing synthetic MR or CT images, and few studies have produced artificial PET images of solid tumors. In addition, to the best of the authors’ knowledge, there is no single study that used GAN-based augmentation techniques to generate synthetic PET images from scratch and by only using mathematical modeling instead of using original PET images that were previously taken by PET scan.

Besides these attempts to augment further artificial medical samples using machine learning tools, a few studies have used biomathematical modeling for producing realistic medical images. For instance, Soltani et al. [24] developed a comprehensive spatiotemporal distribution model to simulate PET 18F-fluorodeoxyglucose (18F-FDG) uptake within the two static and dynamic microvascular networks. Their model potentially investigated the underlying bio-physiological parameters, such as the microvessel conductivity, permeability of transvascular exchange, and interstitial fluid flow fields in both tumor and healthy tissues. In the following, their model is developed by Fasaeiyan et al. [25] via applying the spatiotemporal distribution model to a synthetic tumor microvasculature in order to enhance the quantification and assessment of 18F-FDG uptake on a patient-specific level. The pattern of radiotracer uptake and its accumulation in tissues were similar to experimental observations. Furthermore, Moradi Kashkooli et al. [26] utilized a comprehensive computational model to produce synthetic spatiotemporal snapshots of 18F-FDG uptake on four tumor networks with different sizes but simplified architectures of microvessel growth. They calculated the quantitative and semi-quantitative assessments of a PET radiotracer within tumor networks. They also compared those snapshots with longitudinal PET images of tumor-bearing mice, a pediatric brain tumor, and several other experimental studies, indicating the accuracy of the findings.

In this study, for the first time, we developed a novel computational framework to create synthetic PET images of solid tumors at different stages of growth and angiogenesis, based upon a combination of a multi-scale spatiotemporal distribution model and a machine learning model. This model, unlike many other models that use pre-taken, real PET images as input datasets to generate artificial PET images, produces PET images from scratch using biomathematical modeling. Our model is developed based on the actual bio-physical phenomena behind the 18F-FDG radiotracer accumulation in the tumor and healthy tissues, taking place on different scales. On the cellular scale, tumor-induced angiogenesis is the basis of the model, in which new vasculatures grow from pre-existing vessels to accomplish the tumor’s need for more energy. These new blood vessels act as a source for releasing radiotracer diagnostic agents, here 18F-FDG, into the tissue. At the tissue scale, the model simulates the spatiotemporal distribution of 18F-FDG in both healthy and tumor tissues. Then, a series of images produced by biomathematical modeling is proposed to generate synthetic PET images by a machine learning architecture, here a DCGAN model. The DCGAN model takes the produced 18F-FGD PET images by biomathematical modeling as input and augments more real-like PET samples through the mini-max game theory. The ultimate goal of the present hybrid model is to generate synthesized 18F-FDG PET samples non-invasively to accomplish the need for massive training samples required in the conventional deep learning models.

## 2. Materials and Methods

In this section, first, detailed mathematical models are provided, including the overall theory of tumor-induced angiogenesis modeling and calculating intravascular blood flow, equations governing the fluid flow as well as 18F-FDG radiotracer transport and uptake in tissue and cellular scales. Then, the description of the GAN and DCGAN models, our proposed deep learning model, and its training procedure are provided. Subsequently, the computational domain, grid independence test, and boundary conditions are discussed. Eventually, the solution strategy and model validation are presented.

### 2.1. Tumor-Induced Angiogenesis Modeling

#### 2.1.1. Summary of Mathematical Procedures for Generating Capillary Networks

The present mathematical modeling of tumor-induced angiogenesis is based on a discrete approach that relies on the interaction between endothelial cells (ECs) and their microenvironment. Such discrete angiogenesis models were initially presented by Anderson and Chaplain [27] and then developed by Soltani and Chen [28] by some more realistic architectures of the capillary network. Therefore, in the present study based on our previous works [28,29], we developed an advanced mathematical model of angiogenesis to generate different capillary networks by considering the effect of matrix density and blood flow through vessels, as well as more acceleration of vessels as getting nearer to the tumor.

In summary, once the vascular endothelial growth factor (VEGF) is secreted by hypoxic cells of the tumor, they transport toward the regions with lower VEGF gradients. On the two vertical sides of the computational domain, the parent vessels are taking into account the source of new microvessels. The new-born capillaries begin to move through the domain and be close to the cancerous tissue. The details of rules for sprouting angiogenesis, algorithms, and related governing equations have been discussed in our previous works [26,28,29], but the overall procedure is as follows:

1. Consider the initial conditions of the model;
2. Estimate the probability condition for the motion of every EC by solving a set of probability-based movement algorithms as a function of VEGF concentration in the domain which is discretized through a finite difference scheme based on the previous studies [26,29];
3. Keep each EC stationary or move them left, right, down, or up based on the values calculated in step 2;
4. Check for tip cell fusion (anastomosis). In the discreet mathematical modeling of angiogenesis, when two tip ECs or one tip EC and one stalk EC encounter each other, the anastomosis takes place. In either case, a closed loop is shaped, which allows the blood flows through the new vessels [26,28,29];
5. If anastomosis occurs, only one of the two ECs can continue its migration through the domain;
6. Check for EC branching on its way toward the tumor;
7. If branching occurs, a new EC is included in the list of ECs for the next step;
8. Back to the second step.

#### 2.1.2. Summary of Mathematical Procedures for Intravascular Blood Flow Modeling

The summary of the procedure for calculating intravascular blood flow with adaptable vessels and non-continuous blood behavior is as below:

1. Consider initial guesses for parameters of the model (i.e., intravascular blood pressure (IBP), blood viscosity, hematocrit, and vessel diameter);
2. Calculate IBP in each vascular node iteratively through an algorithm called successive over-relaxation (SOR);
3. Calculate hematocrit and total adaptive stimuli (i.e., wall shear stress stimulus, pressure stimulus of capillaries, and metabolic stimulus);
4. Update capillary diameter based on a new value calculated in step 3;
5. Calculate laminar intravascular blood flow rate according to Poiseuille’s law due to the low Reynolds number [29,30];
6. Calculate the velocity in each capillary;
7. Find hematocrit at the vessel bifurcations;
8. Update the apparent viscosity according to the capillary diameter and hematocrit;
9. Ensure accurate solving by calculating the maximum relative error of IBP and vessel diameter using max xn−xn−1xn−1, in which x can be each of IBP or vessel diameter and n and $\left(n-1\right)$ correspond to the current and previous time steps, respectively;
10. If the maximum relative error is greater than the defined threshold (10^−4^), the iterative procedure should be back to step 2, and the new solutions of parameters should be substituted for the previous quantities. Otherwise, the procedure is considered to be finished, and the IBP values can be used in the next stage of the simulation.

All mathematical formulas governed the vascular blood flow and required parameters for the dynamic adaptive tumor-induced angiogenesis have been discussed in our previous studies [26,28,29].

### 2.2. Interstitial Fluid Flow Modeling

The solid tumor and adjacent healthy tissue can be considered as porous media, based on the literature [31]. The momentum equation for incompressible Newtonian fluid flow within a porous environment at a steady state is simplified to the Darcy equation as follows [31,32]:

(1) $${\vec{V}}_{i}=-\kappa \nabla P_{i}$$

where ${\vec{V}}_{i}$ refers to the interstitial fluid velocity (IFV), $P_{i}$ indicates the interstitial fluid pressure (IFP), and κ is the hydraulic conductivity.

Mass continuity equation for interstitium, taking into account the presence of source and sink terms of mass in biological tissues, is corrected as [31,32]:

(2) $$\nabla .{\vec{V}}_{i}=\phi_{v}-\phi_{l}$$

where $\phi_{v}$ and $\phi_{l}$ are the rate of fluid flow from microvessels to the interstitial space and from interstitial space to lymph vessels, respectively. $\phi_{v}$ and $\phi_{l}$ can be calculated by Starling’s law as [31,32]:

(3) $$\phi_{v}=L_{P}\frac{S}{V}\left(P_{B}-P_{i}-\sigma_{s}\left(\pi_{B}-\pi_{i}\right)\right)$$

(4) $$\phi_{l}=L_{PL}{\left(\frac{S}{V}\right)}_{L}\left(P_{i}-P_{L}\right)$$

in which $\;L_{P}$ is the hydraulic conductivity of the microvessel wall, $\frac{S}{V}$ indicates the microvascular density, $L_{PL}{\left(\frac{S}{V}\right)}_{L}$ is the loss rate due to the lymphatic drainage, $P_{B}$ is the IBP, $\sigma_{s}$ is the coefficient of average osmotic reflection, $\pi_{B}$ is the oncotic pressure of microvessels, $\pi_{i}$ is the oncotic pressure of the interstitial fluid, and $P_{L}$ is the pressure of lymphatic vessels in the healthy tissue. The values of related parameters along with their definitions are presented in Table S1.

### 2.3. Spatiotemporal Modeling of 18F-FDG Transport

In the present work, the transport of 18F-FDG radiotracer is described by a series of partial differential equations (PDEs) instead of ordinary differential equations (ODEs), which have widely been used in conventional compartmental modeling of radiotracer [33]. Using PDEs allowed us to successfully calculate the spatiotemporal distribution of 18F-FDG radiotracer within both healthy and tumor tissues [24,26]. Such mathematical models are also called convection-diffusion-reaction (CDR) equations, which are prevalent in the mathematical modeling of drug delivery to solid tumors [34].

**Figure 1:**
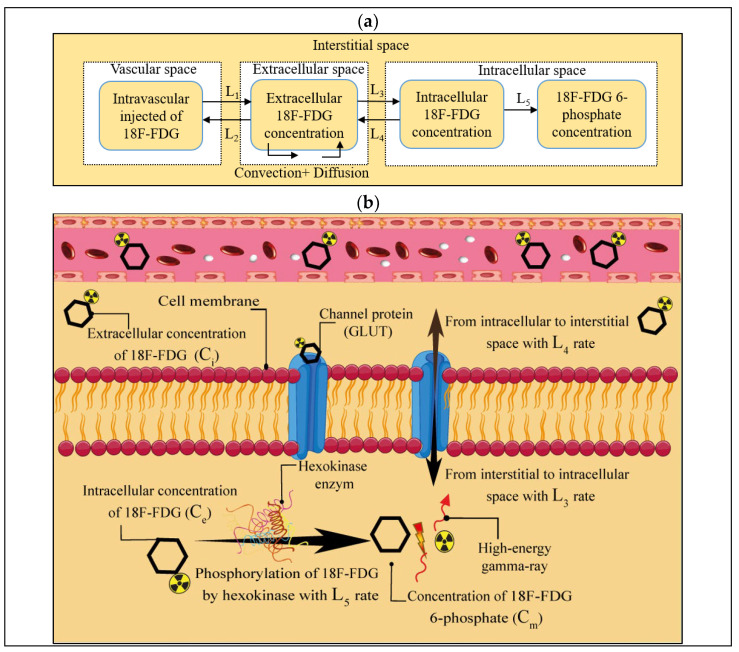
A schematic of the present multi-scale computational model of dynamic transport modeling of the 18F-FDG radiotracer. (**a**) Multi-compartment model of spatiotemporal distribution modeling of 18F-FDG radiotracer. (**b**) Schematic of exchanges of radiotracer between interstitium and microvessels via L_1_ and L_2_ coefficients, transport of extracellular radiotracer in the interstitial space through convection and diffusion mechanisms as well as entering the radiotracer into and out of the cell via L_3_ and L_4_ coefficients, and phosphorylation of intracellularly absorbed radiotracer by hexokinase enzymes via L_5_ constant rate.

Present spatiotemporal modeling of 18F-FDG radiotracer transport measures several biological and physiological phenomena, including radiotracer transport across the microvessels by diffusion and convection mechanisms, radiotracer transport within the interstitial space by diffusion and convection mechanisms, and cell binding and uptake. A schematic of 18F-FDG radiotracer transport from intravascular injection to cellular uptake by tumor cells along with the corresponding compartments is presented in Figure 1. Here, the intravascularly injected 18F-FDG radiotracer is exchanged between the extracellular space and intracellular one and vice versa by glucose transporters (GLUTs), demonstrated by L_3_ and L_4_ constant rates, respectively [26,35,36]. Subsequently, the absorbed 18F-FDG radiotracer may be phosphorylated by hexokinase enzymes to phosphorylated 18F-FDG radiotracer by a constant rate of L_5_, releasing two high-energy gamma rays in opposite directions, which can pass through the body’s lymph drainage system. Then, these high-energy rays can be detected by a PET scan machine. Ultimately, clinicians can detect the tumor tissue state by computer-processing the series of images taken from different angles [26].

#### 2.3.1. Mathematical Formulation of 18F-FDG Radiotracer Transport Modeling

The system of CDR equations for 18F-FDG radiotracer transport modeling is represented as follows [24,26]:

(5) $$\frac{\partial C_{i}}{\partial t}=-{\vec{V}}_{i}\nabla C_{i}+D_{eff}\nabla^{2}C_{i}-L_{3}C_{i}+L_{4}C_{e}+\left(\Phi_{V}-\Phi_{L}\right)$$

(6) $$\frac{\partial C_{e}}{\partial t}=L_{3}C_{i}-L_{4}C_{e}-L_{5}C_{e}$$

(7) $$\frac{\partial C_{m}}{\partial t}=L_{5}C_{e}$$

(8) $$L_{1}=\phi_{v}\left(1-\sigma_{f}\right)-\frac{P_{m}S}{V}\frac{Pe}{\exp\left(Pe\right)-1}$$

(9) $$L_{2}=\frac{P_{m}S}{V}\frac{Pe}{\exp\left(Pe\right)-1}+\phi_{l}$$

(10) $$Pe=\frac{\phi_{v}\left(1-\sigma_{f}\right)}{P_{m}\frac{S}{V}}$$

where $C_{i}$ is the extracellular 18F-FDG radiotracer concentration normalized to extracellular volume, $C_{e}$ is the intracellular concentration of 18F-FDG radiotracer, and $C_{m}$ is the phosphorylated intracellular concentration of 18F-FDG radiotracer. $L_{3}$, $L_{4}$, and $L_{5}$ are transport rate constants between the extracellular matrix (ECM) and tumor cell, the transport rate from tumor cell to the ECM, and the 18F-FDG radiotracer phosphorylation rate, respectively. Furthermore, $L_{1}$ and $L_{2}$ are the exchange rate parameters between plasma and ECM,$\;P_{m}$ is the vascular permeability coefficient, $\sigma_{f}$ is the filtration reflection coefficient and Pe is the Peclet number.

In Equation (5), $\Phi_{V}$ and $\Phi_{L}$ are the rate of radiotracer transport per unit volume from microvessels into the interstitial space and from the interstitial space into the lymphatic drainage system, respectively. These two parameters are expressed based on Patlak’s model as [37,38]:

(11) $$\Phi_{V}=\phi_{v}\left(1-\sigma_{f}\right)C_{p}+P_{m}\frac{S}{V}\left(C_{p}-C_{i}\right)\frac{Pe}{\exp\left(Pe\right)-1}$$

(12) $$\Phi_{L}=\phi_{l}C_{i}$$

in which $C_{p}$ is the 18F-FDG radiotracer plasma arterial concentration [39]. The values of 18F-FDG radiotracer transport parameters along with their definitions are provided in Table S2.

#### 2.3.2. Semi-Quantitative Assessment of 18F-FDG Radiotracer Transport Modeling

For quantitative analysis of radiotracer uptake in different stages of tumor progression, the standardized uptake value (SUV) index is measured in clinical oncological practice. SUV is determined as the ratio of the total tissue radioactivity concentration investigated in a region of interest to the radioactivity injected into the body, normalized by the body weight as [26]:

(13) $$\mathrm{SUV}=\frac{\mathrm{Tissue}\;\mathrm{radioactivity}\;\mathrm{concentration}\;\left(C_{total}\right)}{\mathrm{Injected}\;\mathrm{radioactivity}}\times \mathrm{Body}\;\mathrm{weight}$$

(14) $$C_{total}=C_{i}+C_{e}+C_{m}$$

A patient with 75 kg weight is considered for further analysis [39]. Additionally, the total 18F-FDG radiotracer concentration ($C_{total}$) is determined as a sum of three other concentrations ($C_{i}$, $C_{e}$, and $C_{m}$).

### 2.4. Generative Adversarial Network

GAN is a novel deep learning architecture that was invented by Goodfellow et al. [9]. The original GAN model consists of two phases: generative G and discriminator D neural networks. The generative model aims to produce synthetic examples from a random distribution. The discriminative model reviews the generated data to evaluate the probability that the sample is drawn from the actual training dataset or the generative model. The generative and discriminative models at the same time compete with one against the other to become trained by using the minimax game theory. The training procedure goal for G is to maximize the probability of D making mistakes [9].

Generator $G\left(z,\theta_{g}\right)$ is a differentiable function demonstrated by a multilayer perceptron that shows a mapping to the data space with parameters $\;\theta_{g}$. A prior distribution $\rho_{z}$ is determined on random noise variables z to learn the generator’s distribution $\rho_{g}$ over the data input space x. The discriminator $D\left(x,\theta_{d}\right)$, also a multilayer perceptron, takes the synthetic dataset generated by G as well as the real training dataset and results in a single scalar value. During the training process, the discriminator D tries to maximize $\log\left(D\left(x\right)\right)+\log\left(1-D\left(G\left(z\right)\right)\right)$ in which $D\left(x\right)$ is the probability of the examples being real and $D\left(G\left(z\right)\right)$ is the probability of them having been produced from $G\left(z\right)$. Simultaneously, G is trained to minimize the cross-entropy loss function represented by $\log\left(1-D\left(G\left(z\right)\right)\right)$ and thus successively learn to generate data samples so that D cannot distinguish them from the actual dataset. This is thus a two-player minimax optimization game between generator and discriminator with the following value function $\;V\left(D,\;G\right)$ [9]:

(15) $$\underset{G}{\min}\underset{D}{\max}V\left(D,\;G\right)=E_{x\sim \rho_{data\left(x\right)}}\left[\log\left(D\left(x\right)\right)\right]+E_{z\sim \rho_{data\left(z\right)}}\left[\log\left(1-D\left(G\left(z\right)\right)\right)\right]$$

where E indicates a statistical expectation, z is the input noise vector, and x is the real image dataset. $\rho_{data\left(x\right)}$ and $\rho_{data\left(z\right)}$ stand for the distribution of the real data and the random noise, respectively. The training process of a GAN model is composed of sampling mini-batches of the real training data and data generated by the generator’s distribution $\rho_{g}$ and updating the discriminator’s parameter $\theta_{d}$ by stochastic gradient ascent of $\log\left(D\left(x\right)\right)+\log\left(1-D\left(G\left(z\right)\right)\right)$, followed by sampling mini-batches of data points from the distribution $\rho_{z}$ and updating the generator’s parameters $\;\theta_{g}$ through stochastic gradient descent of $\log\left(1-D\left(G\left(z\right)\right)\right)$ until an optimum is reached [9].

#### 2.4.1. Deep Convolutional Generative Adversarial Network

DCGANs [40] are convolutional networks that have fewer constraints than the architectural topology of the first GAN [9] and convolutional GANs. DCGANs have more stability during the training and are able to generate higher resolution output images. To produce synthetic images via the DCGAN model, there will be two stages: a learning stage and a generation stage. During the learning stage, the generator model produces samples from an input N-dimensional uniform distribution vector and operates on this vector via successive upsampling operations, ultimately producing fake samples from it. The discriminator tries to differentiate between fake images generated by the generator model and real images from the training dataset [40].

The DCGAN replaces all max-pooling layers with stride convolutions (discriminator) and proposes fractional-strided convolutions for upsampling (generator). It also uses batch norm in generator and discriminator for adjustment of extracted feature scale and also excludes fully connected hidden layers. In addition, the leaky rectified linear unit (Leaky ReLU) activation function is used in all layers of the discriminator. The ReLU functions are used for all of generator’s layers except for the output layer, which uses the *Tanh* activation function [40].

#### 2.4.2. Proposed GAN Model

**Figure 2:**
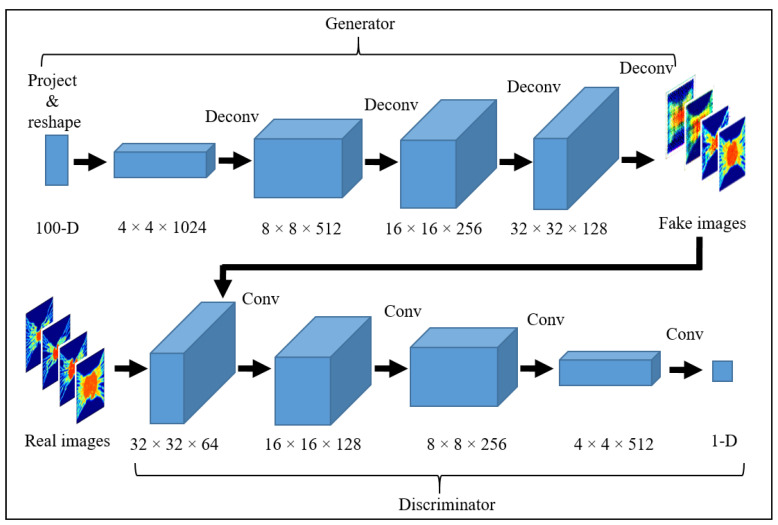
Generator and discriminator architectures are used to generate synthetic or fake 18F-FDG PET images. A series of four fractionally-strided convolutions (i.e., deconvolutions) as a generator to convert a vector of random 100-dimensional uniform distribution into a 64 × 64 × 3 pixel image, and then the discriminator takes these generated images and 1700 input images of size 64 × 64 × 3, which are generated previously using biomathematical modeling, and calculates the likelihood probability (0, 1) score values using four convolution layers to distinguish the synthetic and real 18F-FDG PET images.

In every model, synthetic images should be generated with the desired settings to adopt DCGAN for medical purposes. The architecture for the proposed synthetic PET image GAN model is shown in Figure 2. The generator input is a vector of random 100-dimensional uniform distribution, and the output is an 18F-FGD PET image of size 64 × 64 × 3. The present network utilizes fully connected layers, and the generator is comprised of four fractionally-strided convolutions (i.e., deconvolutions) with 2D batch normalization layers. A ReLU activation function was applied to all layers except for the last layer, which uses the Tanh function. The discriminator model takes an image of size 64 × 64 × 3 (18F-FGD PET images generated using biomathematical modeling) as input and decides whether it belongs to the real training data or not (1 for a real image and 0 for a fake image). The discriminator network includes 4 convolution layers with a kernel size of 4 × 4. Each convolutional layer uses strided convolutions to decrease the spatial dimensionality instead of using pooling layers. Batch normalization and Leaky ReLU activation function are used for each convolutional layer of the discriminator except the output layer. A Sigmoid function is applied in the output layer to compute the likelihood probability (0, 1) score.

#### 2.4.3. Training Procedure

**Figure 3:**
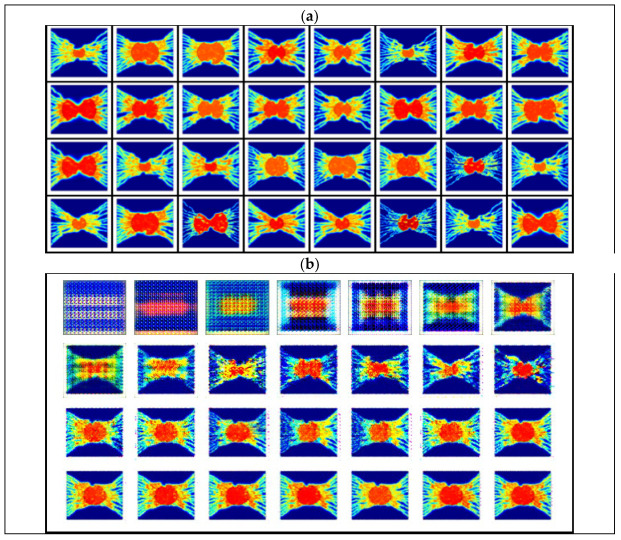
Thirty-two samples of input images are given to the DCGAN model and synthetically generated output images. (**a**) Sample of input images as training data set consisting of 1700 images of the spatiotemporal distribution of SUV at various times post 18F-FDG injection in 10 different tumor sizes, ranging from 1 to 3 cm diameter. (**b**) Visualization of the DCGAN output images after different epochs of training.

The generator and discriminator models are trained through an iterative training flow with mini-batches of 50 image samples. A random 100-dimensional vector from a uniform distribution value range between [–1, 11] is given as an input for the generator model. All weights are initialized from a zero-centered normal distribution with a standard deviation of 0.02. In all models, the slope of the Leaky ReLU is set to 0.2, and stochastic gradient descent is applied during the training procedure with the Adam optimizer. The first momentum term of the optimizer is set to $\beta_{1}\;$= 0.5 to stabilize training, and the second one is set to $\beta_{2}\;$= 0.999. Ultimately, a learning rate of 0.0001 is applied during the training process. Figure 3 shows a sample of the input images and the output images after different steps.

#### 2.4.4. Quantitative Evaluation of Synthesized Generated 18F-FGD PET Images

To analyze the performance of generated PET images quantitatively, two widely used metrics are calculated including peak signal-to-noise ratio (PSNR) and structural similarity index measure (SSIM). The PSNR measures the ratio between the highest possible intensity value and the mean squared error of the augmented and real images. A higher PSNR quantity indicates a higher image quality. The output images of the biomathematical model without implementing the DCGAN and generated PET images using the DCGAN model are separately compared with each other and previously published experimental results of 18F-FDG PET images. The proposed quality index is defined as [41]:

(16) $$\mathrm{PSNR}=10\log\frac{{\left(\max\left(y\right)\right)}^{2}}{\frac{1}{n}\sum_{i}^{n}{\left(y_{i}-{\hat{y}}_{i}\right)}^{2}}\;$$

where n is the overall pixels of the image, $y_{i}$ and ${\hat{y}}_{i}\;$ are the *i*th pixel in the original and the generated images, respectively. SSIM measures the inter similarities between pixels of two images, i.e., yields the index of similarity in the pixel values of two given images [42]. In our study, an SSIM index of 0 indicates no similarity, and 1 indicates total positive similarity [42]:

(17) $$\mathrm{SSIM}\left(x,y\right)=\frac{\left(2\mu_{x}\mu_{y}+C_{1}\right)+\left(2\sigma_{\mathrm{xy}}+C_{2}\right)}{\left(\mu_{x}{}^{2}+\mu_{y}{}^{2}+C_{1}\right)\left(\sigma_{x}{}^{2}+\sigma_{y}{}^{2}+C_{2}\right)}$$

in which  x and  y represent two generated and ground truth PET images, respectively. The averages of x is $\mu_{x}$ and the averages of y is $\mu_{y}$.${\;\sigma }_{x}$ is the variance of x, $\sigma_{y}$ is the variance of  y, and $\sigma_{\mathrm{xy}}$ denotes the covariance of y and x. To stabilize division, $C_{1}={\left(K_{1}L\right)}^{2}$ and $C_{2}={\left(K_{2}L\right)}^{2}$ are proposed in which  L is the dynamic range of the pixel values, $K_{1}=0.01$ and $K_{2}=0.03$.

### 2.5. Computational Domain and Boundary Conditions

In this work, a two-dimensional circular solid tumor is considered with 10 various diameters, ranging from 1 cm to 3 cm. Such ranges of tumor and corresponding capillary networks reveal the different stages of tumor progression and angiogenesis. All the considered boundary conditions are presented in Table S3 and Figure S2. The grid size for solving Darcy’s law, conservations of mass and momentum, as well as CDR equations had roughly 29,000 triangular elements, of which approximately 550 were edge elements, with minimum element quality of 0.64 and average element quality of 0.90. To ensure that the results are relatively insensitive to the grid size, four other courser grids are tested, but no significant change, less than 3%, in 18F-FDG radiotracer concentration and IFP profiles is observed.

### 2.6. Solution Strategy

**Figure 4:**
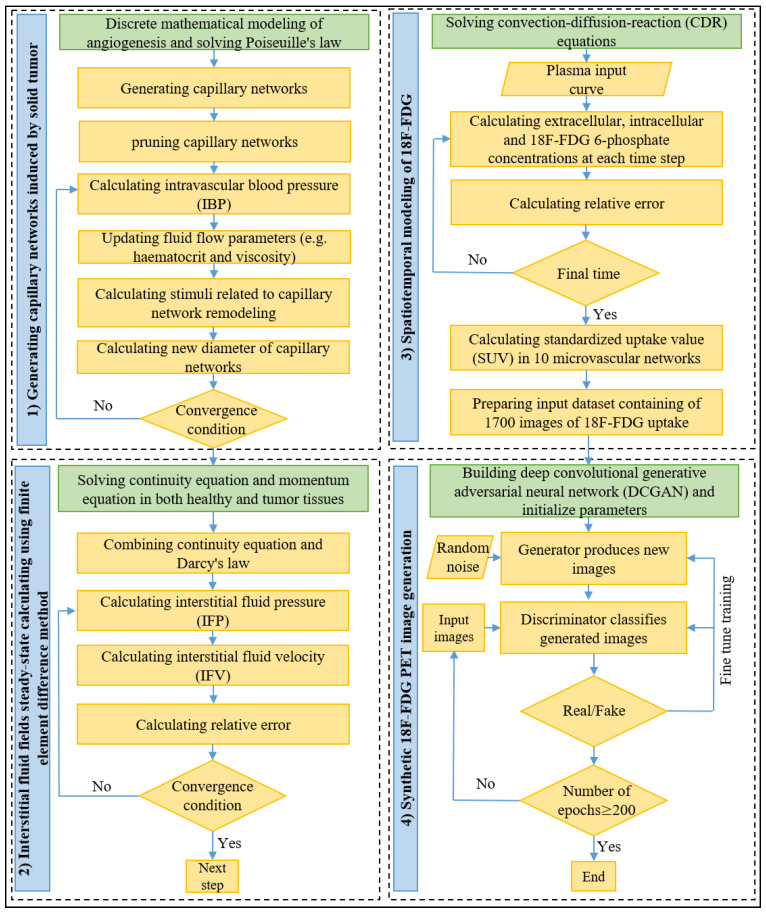
Block diagram showing step-by-step approach used to generate synthetic 18F-FDG PET images. Further details are presented in the solution strategy section.

As shown in Figure 4, the presented computational framework includes four main parts:

1. Generation of tumor-associated vasculatures and calculating vascular blood flow through microvessels;
2. Calculating interstitial fluid flow parameters in both tumor and healthy tissues;
3. Calculating the spatiotemporal distribution of 18F-FDG radiotracer in generated capillary networks;
4. Generating synthetic 18F-FDG PET images using DCGAN.

Firstly, the probability of movement of each EC is calculated, and then the anastomosis and branching are checked using the approach introduced by Soltani [29] to generate capillary networks. In the following, the intravascular blood flow with non-continuous behavior of blood through capillaries with adaptable microvessels is solved iteratively. Secondly, the calculated IBP in the previous stage is imported and used to calculate IFV and IFP by solving Equations (1)–(4). Thirdly, 18F-FDG plasma arterial concentration, as well as calculated interstitial pressure and velocity values, is used to solve coupled CDR equations, represented in Equations (5)–(12). Different intracellular and extracellular 18F-FDG radiotracer concentrations are used to calculate SUV, according to Equation (13). Subsequently, the SUV is normalized to make all the values in the dataset lie between 0 and 3, thus bringing them to a common scale. In each capillary network, an image of SUV distribution is extracted every 20 s, while the total simulation time for solving the CDR equation is considered 1 h, as defined by Boellaard et al. [43]. Since in the first ten images (images taken between [0 s, 180 s]), 18F-FDG has only accumulated nearby the capillary network, these images are excluded, resulting in 170 images in each network. We repeat the same process for all 10 capillary networks, and thus a series of 10 × 170 = 1700 images is prepared to be implemented in the next stages of the presented framework. Eventually, in the unsupervised machine learning stage, the DCGAN architecture, which consists of two generator and discriminator models, is used to produce high-quality artificial PET images during the training using the formerly prepared dataset.

The governing equations related to tumor-induced angiogenesis and intravascular blood flow are discretized using Euler finite difference method and are solved by C++ programming language, while the governing equations related to the interstitial fluid flow and spatiotemporal modeling of 18F-FDG radiotracer concentration are solved by the commercial computational fluid dynamics software COMSOL Multiphysics 5.5 (COMSOL, Inc., Burlington, MA, USA), which works based on the finite element method. The direct solver of multifrontal massively parallel sparse direct solver (MUMPS) is considered with the backward differentiation formula (BDF) time-stepping method. Relative tolerance of 0.001 and a time step of 10 s are considered. It should be noted that smaller time steps did not considerably change the results but increased the computational cost. In addition, the training process of the DCGAN is implemented by PyTorch 1.9 (High-Performance Deep Learning Library, New York, NY, USA), Python 3.9 (Python Software Foundation, Wilmington, DE, USA) and is performed for the training of 200 epochs. All of these computational simulations are carried out on a personal laptop by CPU Intel (R) Core i5-6200U processor CPU 2.4 GHz with memory 8 Gbytes.

### 2.7. Model Validation

In our previous works [26,29], we have confirmed the accuracy and validity of the architecture of the generated capillary networks as well as their IBP values by comparing the qualitative and quantitative results against both experimental data and numerical results. The accuracy of solving the Darcy equation and conservation of mass in interstitium for calculating the interstitial fluid flow parameters (IFV and IFP) and their distribution are extensively compared against numerical predictions and experimental data of previously published studies, showing excellent agreement. In addition, to verify the accurate solving of the CDR equations regarding spatiotemporal modeling of the 18F-FDG radiotracer, the total concentration of 18F-FDG in solid tumor has been compared with in vivo results of Backes et al. [39], indicating very good compatibility. These different validations of the presented biomathematical model are not presented here for brevity, but readers are referred to the literature [26,29] for more information. However, we have compared and discussed the results in a random tumor network with experimental and numerical predictions in the following section.

## 3. Results and Discussion

In the present study, a robust, comprehensive, multi-scale framework is developed, which consists of two phases: biomathematical modeling and an unsupervised machine learning architecture model. The ultimate goal of the framework is to generate synthetic 18F-FDG radiotracer PET images in different stages of solid tumor progression and angiogenesis. The biomathematical modeling phase simulates the real bio-physical phenomena behind the tumor-induced angiogenesis, intravascular and interstitial fluid flow, as well as 18F-FDG radiotracer distribution in both tumor and healthy tissues. These biological phenomena occur on various scales, ranging from cellular to tissue scales. Ultimately, the results of this stage are used to create a series of 1700 images including spatiotemporal distribution of SUVs in 10 different and unique microvascular networks, which is used in the next phase. The machine learning phase includes employing the DCGAN model, which has two generator and discriminatory models. These two models simultaneously compete with each other to generate high-quality artificial PET images. To the best of our knowledge, this study is the first to augment synthetic PET images of solid tumors using samples generated by biomathematical modeling of 18F-FDG radiotracer uptake. In the fowling, the results of these two phases will be presented and discussed in detail.

**Figure 5:**
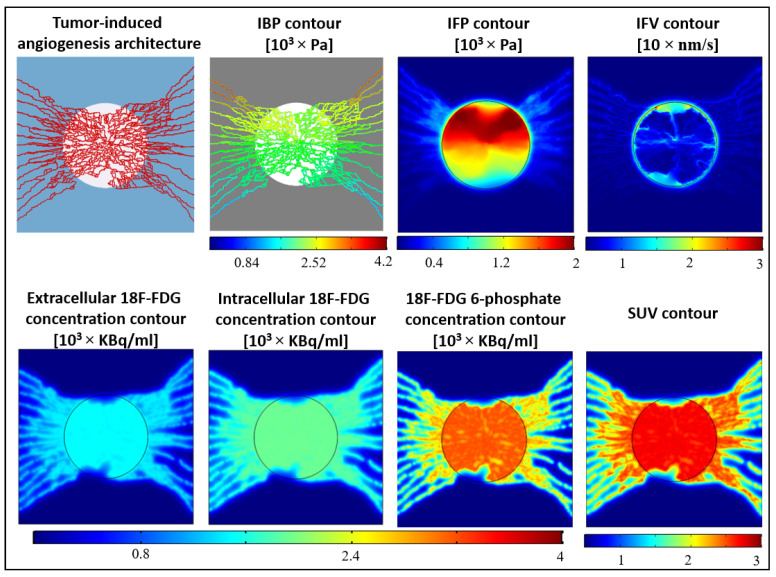
Results of biomathematical modeling in a tumor capillary network with 2.4 cm diameter; tumor-induced angiogenesis architecture, intravascular and interstitial fluid flow parameters, as well as local distribution of 18F-FDG radiotracer uptake in both tumor and healthy tissues one-hour post-injection.

Since our main focus in the present study is on the synthesize 18F-FDG PET image generated by the GAN model, the results of biomathematical modeling are merely shown for a random capillary network of a 2.4 cm tumor diameter as a representative of the rest of the microvascular networks. However, we have comprehensively discussed different details of angiogenesis as well as transport and uptake of 18F-FDG radiotracer in our previous works [24,26]. Figure 5 shows the results of biomathematical modeling in different stages, performed to create the input dataset of DCGAN.

The backbone of angiogenesis on a cellular scale is characterized by the interaction of ECs and ECM. During angiogenesis, the blood microvessels move from the parent vessel on the two side edges of the domain into a circular tumor at the center of the domain. From a biological viewpoint, the main reason for this movement towards the tumor is the higher VEGF gradient within the tumor tissue released by hypoxic cancer cells [27,44]. Six different stages of angiogenesis are illustrated in Figure S1. In the first days of starting angiogenesis, new capillaries grow from the parent vessels and then they branch and make new, tree-like, and more matured vessels. Subsequently, as the microvessels are approaching the tumor, the density of microvessels and their tortuosity increase. This process continues until the new-born microvessels reach the borders of tumor tissue and then the avascular tumor develops to its fatal vascular phase of growth [45]. The two first panels in Figure 5 show the final, pruned capillaries carrying blood and their IBP distributions. The IBP near the inlet has the highest magnitudes (see Figure S2a for intravascular boundary conditions). However, the IBP in the network is 3030.41 ± 2098.66 Pa (mean ± standard deviation), which is in good agreement with the reported physiological observations [38] (i.e., 2660–3333 Pa) and the results of numerical studies [28,46].

The two latter panels in the first row of Figure 5 illustrate the distribution of interstitial fluid flow parameters. As expected, the tumor tissue has the greatest IFP through the domain with an average of 2378.40 Pa, indicating the hallmark of solid tumors [47]. IFP values decrease sharply in the periphery of the tumor with an average value of 182.70 Pa. The main reason for interstitial hypertension is the lack of an efficient lymphatic drainage system in the tumor tissue as well as high leaky blood microvessels [37,48]. Consequently, the direct result of such elevated IFP is reducing blood flow and therefore insufficient and poor delivery of therapeutic and diagnostic agents into the central zones of the tumor [26,48,49]. In contrast, the IFV has a very low magnitude over the domain, with an average value of 1.47 × 10^−7^ m/s, unless at the tumor periphery, where a high-pressure gradient has existed. Thus, according to Darcy’s law (see Equation (1)), the highest IFV takes place at the tumor border. These results have also been reported in other experimental [50,51,52] and numerical [25,28,31,53,54] studies.

The panels in the second row of Figure 5 illustrate the extracellular, intracellular, and phosphated18F-FDG radiotracer concentrations one-hour post-injection. The tumor tissue in each contour can be easily distinguished from the surrounding healthy tissue by drastically accumulated 18F-FDG radiotracer. The 6-phosphate18F-FDG concentration in the tumor and healthy tissues has an average value of 300.12 and 85.34 kBq/mL, respectively. The reason for such elevated radiotracer concentration in the tumor is that tumors exhibit a greater reliance on glycolysis for glucose metabolism and energy production than surrounding healthy tissues, a phenomenon known as the Warburg effect [35]. Therefore, this principal discriminator of tumors from healthy tissues by 18F-FDG PET imaging is utilized for the detection of metastatic cancer and as a metric of response to therapy in clinical trials [55]. As indicated in Equations (1) and (3), SUV distribution has a similar spatial trend to the distribution of phosphorylated 18F-FDG concentration. In this capillary network, the tumor and healthy tissues have average SUV values of 2.49 and 0.84, respectively. These calculated SUVs are in excellent agreement with the quantitative experimental analysis of Al-Nabhani et al. [56] and Sha et al. [57] in whole-body PET imaging and tumor-bearing mice, respectively. Additionally, Video S1 illustrates the spatiotemporal changes in SUV distribution in the capillary network. As shown in the video, owing to the high permeability of tumor-associated vasculatures, the maximum radiotracer concentration occurs in the tumor area at all time frames. However, since the microvessels in the capillary network act as source terms for releasing radiotracer, at the initial times post-injection, the 18F-FDG radiotracer concentration is mainly accumulated nearby the capillary walls [26]. The 18F-FDG radiotracer is then transported and distributed in the other areas of the tissue through the diffusion and convection mechanisms.

**Figure 6:**
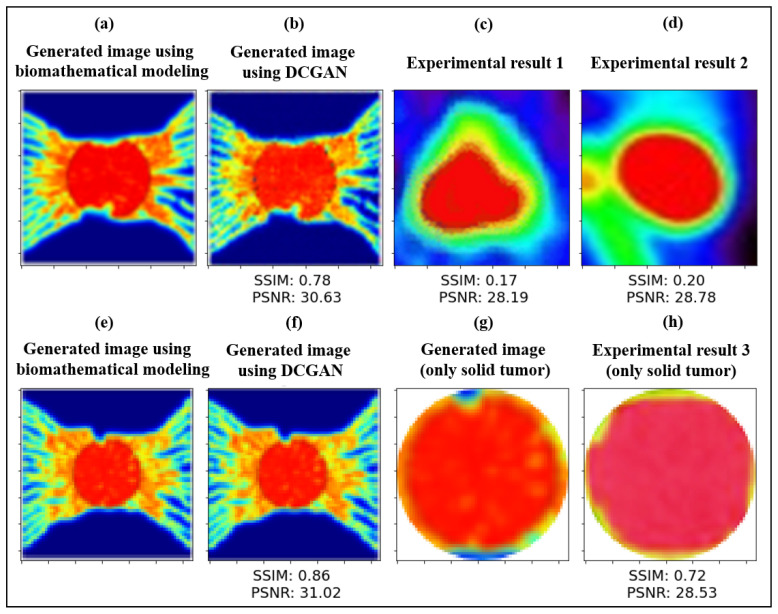
Quantitative and qualitative comparisons of the synthetic and clinical 18F-FDG PET radiotracer images of solid tumors at different stages of progression and angiogenesis. (**a**) A synthetic PET image generated by biomathematical modeling for a tumor with 2.4 cm diameter is compared with (**b**) an augmented PET image generated using the DCGAN model, and (**c**,**d**) two clinical PET images [58,57]. Comparison between (**e**) a synthetic PET image generated by biomathematical modeling without implementing the DCGAN for a 2cm tumor and (**f**) another augmented PET image generated using DCGAN. (**g**) The solid tumor in panel (**f**) is also compared with (**h**) another image of solid tumor taken experimentally [59], showing excellent agreement in terms of quantitative assessments with SSIM and PSNR values and qualitative comparison of radiotracer uptake in solid tumor. SSIM and PSNR values are defined according to Equations (16) and (17), respectively. Images in panels (**c**,**d**,**h**) are adopted and printed with permission from [57,58,59]. Copyright 2022.

The mean PSNR value of images generated using biomathematical modeling only and the DCGAN model is 30.82 (i.e., 30.63 for a 2.4-cm tumor and 31.02 for a 2-cm tumor), as indicated in Figure 6a,b,e,f. The mean SSSIM value of images generated by biomathematical modeling only and the DCGAN model is 0.82 (i.e., 0.78 for a 2.4-cm tumor and 0.86 for a 2-cm tumor). These results demonstrate the supreme quality of augmented PET images using a GAN-based model from the given input PET images, which are created by biomathematical modeling.

As shown in Figure 6c,d, the PSNR and SSIM values of the generated PET image and a pediatric brain tumor [58] are 28.19 and 0.17, respectively. Furthermore, applying these quantitative assessments on a longitudinal PET image of a U87 implanted in the shoulder region of a mouse [57] results in 28.78 and 0.20 for PSNR and SSIM, respectively. Given the complexity of the bio-physical properties of the tumor microenvironment, the exceptional quantitative and qualitative agreement achieved is a testament to the level of sophistication in the experimental PET images and the potential of our presented hybrid framework. Nevertheless, small discrepancies between the computational and experimental quantitative metrics are attributed to the location and size of the tumor and surrounding healthy tissues. We have also measured the qualitative evaluation of an augmented PET image generated by the DCGAN model and another solid tumor with the same order of size in breast lesion [59]. As indicated in Figure 6g,h, the results are only shown for solid tumors, which have been cut circularly. The PSNR and SSIM values are 28.53 and 0.72, respectively. These quantitative similarities are consistent with other GAN-based synthetic medical image generation models [11,18,19,20,21,23], which used real clinical medical images as the training dataset.

To the best of our knowledge, there is no single study that addresses what criteria for each image quality metric (e.g., SSIM, PSNR, inception score, and frechet inception distance) in the GAN-based augmentation models are sufficient enough to fool the clinicians into whether the medical images are taken experimentally or generated synthetically. However, some recent studies have benefited the human assessment as a gold standard to compare the synthesized medical samples with original medical images [12,60,61,62]. Thus, a potential perspective work would be conducted a visual assessment by some blinded radiologists to identify real 18F-FDG PET images versus augmented ones and rate them to find out what range for these quantitative metrics is sufficient enough as a diagnostic criterion.

A specific point that should be noted in Figure 6c,d is that some differences in the microenvironment details nearby the boundary of tumor tissue between our generated PET samples and real clinical PET images are due to the fact that such heterogeneities cannot be detected during routine PET imaging. This is mainly because of the limited scale size of PET scans, possible voluntary movements by patients, and several resolutions degrading phenomena (e.g., inter-crystal blurring) that can eventually decrease the quality of PET images [26,63]. Another point worth mentioning is that due to the complexity of the TME, it cannot be extensively modeled using a set of PDEs with all biophysiological details. For instance, in a real scenario of tumor-induced angiogenesis, the ECs may move towards different sides from several parent vessels located on every side of tumor tissue, while our model only considers the movement of ECs from two vertical parent vessels. In any case, the heterogeneity models, such as the one developed herein, can help the clinicians and cancer researchers to understand the biophysics of TME in a more depth way. Furthermore, with ongoing research toward higher-resolution PET images, it is predicted that more complex heterogeneities of radiopharmaceutical uptakes can be captured in the next-generation PET imaging [26].

## 4. Conclusions

The limited dataset is a common challenge in deep learning-based networks in medical image analysis. In this context, GAN-based models can synthesize realistic further training samples to fill the medical image lack in the real clinical image. In the present study, a novel, comprehensive, and robust framework incorporating both biomathematical modeling and machine learning tools are introduced to generate synthetic PET images. The biomathematical modeling includes a series of equations for generating tumor-induced capillary network, calculating intravascular blood flow, interstitial fluid flow, and spatiotemporal distribution of 18F-FDG radiotracer uptake. The DCGAN model, an unsupervised machine learning method, consists of the generator and discriminator to produce new synthetic 18F-FDG radiotracer images from 170 images in each of 10 generated tumor capillary networks.

The capillary network, IBP, IFV, IFP, as well as intracellular and extracellular 18F-FDG radiotracer concentrations are calculated and extensively compared with previously-published computational and experimental data, illustrating a very good agreement. Subsequently, for the first time, instead of using clinical detests of PET images, a series of 1700 PET images created by biomathematical modeling is used as the input dataset for the DCGAN model to generate augmented PET images. Very similar qualitative and quantitative spatiotemporal distributions of 18F-FDG radiotracer uptake can be observed in both generated images by biomathematical modeling and the GAN-based model with the real clinical PET images. These similarities demonstrate the robustness of the present model in simulating the complex biological phenomena behind 18F-FDG radiotracer transport, distribution, and cellular uptake. A comparison between generated images using DCGAN and biomathematical modeling shows a mean SSIM value of 0.82 and PSNR value of 30.82. In addition, the quantitative similarity between the generated image by the DCGAN model and the real clinical PET image is remarkable as indicated by the excellent agreement between those pixel images, with the SSIM value of 0.72 and PSNR value of 28.53.

The combined model presented herein can be generalized in other tumor-associated vasculatures using tumor capillary networks taken from clinical images in different scales, such as skin fold window chamber, histology imaging, micro-CT, etc. [44]. Additionally, the model can be developed to simulate other radiopharmaceuticals such as 68Ga-PSMA-11 and 18F-FMISO, which can ultimately help to supplement the training medical image dataset. In order to achieve higher quality, stability, and variation of output samples of the GAN-based model, the PGGAN [64] algorithm will be implemented in our future works.

## Data Availability

All datasets and computational codes presented in this study are available on reasonable request from the corresponding author.

## Supplementary Materials

The following supporting information can be downloaded at: https://www.mdpi.com/article/10.3390/cancers14112786/s1, Table S1: Parameters used for interstitial transport modeling, Table S2: Parameters used for spatiotemporal distribution modeling of 18F-FDG transport, Table S3: Boundary conditions of computational modelings, Figure S1: Results of discrete mathematical modeling of tumor-induced angiogenesis for six different stages of capillary networks, Figure S2: Schematic of the computational domain and implemented boundary conditions, Video S1: SUV distribution in a solid tumor with 2.4 cm diameter at different times post-injection. References [26,31] are cited in the Supplementary Materials.
