# Supplementary material for: Synthetic 18F-FDG PET Image Generation Using a Combination of Biomathematical Modeling and Machine Learning
Source: Cancers (Basel). 2022 Jun 3;14(11):2786. doi: 10.3390/cancers14112786 (PMC9179454; doi:10.3390/cancers14112786)
Supplement: Supplementary file 1 [file cancers-14-02786-s001.zip › cancers-1719327-supplementary.pdf]

cancers MDPI

Supplementary Materials

Synthetic 18F-FDG PET Image Generation using a Combination of Biomathematical Modeling and Machine Learning

Mohammad Amin Abazari , Madjid Soltani , Farshad Moradi Kashkooli and Kaamran Raahemifar

Table S1. Parameters used for interstitial transport modeling.

Parameter	Unit	Definition	Tissue type	Value	Reference
π_i	mmHg	Oncotic pressure of interstitial fluid	Tumor	10	[26]
			Healthy	15	
π_B	mmHg	Oncotic pressure of microvessels	Tumor	20	[26, 31]
			Healthy	20	
σ_s	-	Coefficient of average osmotic reflection	Tumor	0.91	[26, 31]
			Healthy	0.82	
L_P	$\frac{m}{Pa.s}$	Hydraulic conductivity of the microvessel wall	Tumor	2.1×10^{-11}	[26, 31]
			Healthy	0.27×10^{-11}	
$L_{PL}(\frac{S}{V})_L$	$\frac{1}{Pa.s}$	Coefficient of lymph filtration	Healthy	1×10^{-7}	[26]
κ	$\frac{cm^2}{mmHg.s}$	Hydraulic conductivity of interstitium	Tumor	4.13×10^{-8}	[26, 31]
			Healthy	8.53×10^{-9}	
P_L	Pa	Hydrostatic pressure of lymphatic vessels	Healthy	0	[26, 31]

Table S2. Parameters used for spatiotemporal distribution modeling of 18F-FDG transport.

Parameter	Unit	Definition	Tissue type	Value	Reference
D_{eff}	$\frac{m^2}{s}$	Effective diffusion coefficient	Tumor	1.23×10^{-9}	[26]
			Healthy	0.37×10^{-9}	
P_m	$\frac{m}{s}$	Microvessel permeability coefficient	Tumor	7.83×10^{-6}	[26]
			Healthy	2.26×10^{-6}	
σ_f	-	Filtration reflection coefficient		0.9	[26]
L_3	$\frac{1}{s}$	Transport rate parameter into the cell		8.2×10^{-4}	[26]
L_4	$\frac{1}{s}$	Transport rate parameter out of the cell		6.7×10^{-4}	[26]
L_5	$\frac{1}{s}$	Phosphorylation rate		5.3×10^{-4}	[26]

Table S3. Boundary conditions of computational modelings.

Regions	Boundary conditions	
	**Interstitial fluid flow**	**18F-FDG transport modeling**
Inner boundary	$(-\kappa_t P_i\|_{\Omega^t}) = (-\kappa_n P_i\|_{\Omega^n})$<br>$(P_i\|_{\Omega^t}) = (P_i\|_{\Omega^n})$	$\left((D_{eff}{}^{t}\nabla C + V_i C)C\|_{\Omega^t}\right) = \left((D_{eff}{}^{n}\nabla C + V_i C)C\|_{\Omega^n}\right)$<br>$(C\|_{\Omega^t}) = (C\|_{\Omega^n})$
Outer boundary	$P_i = \text{Constant}$	$-\text{n}.\nabla C = 0$

Ω^t and Ω^n indicate the tumor and healthy tissues at their boundaries, respectively. P_i and C are interstitial fluid pressure and radiotracer concentration, respectively.

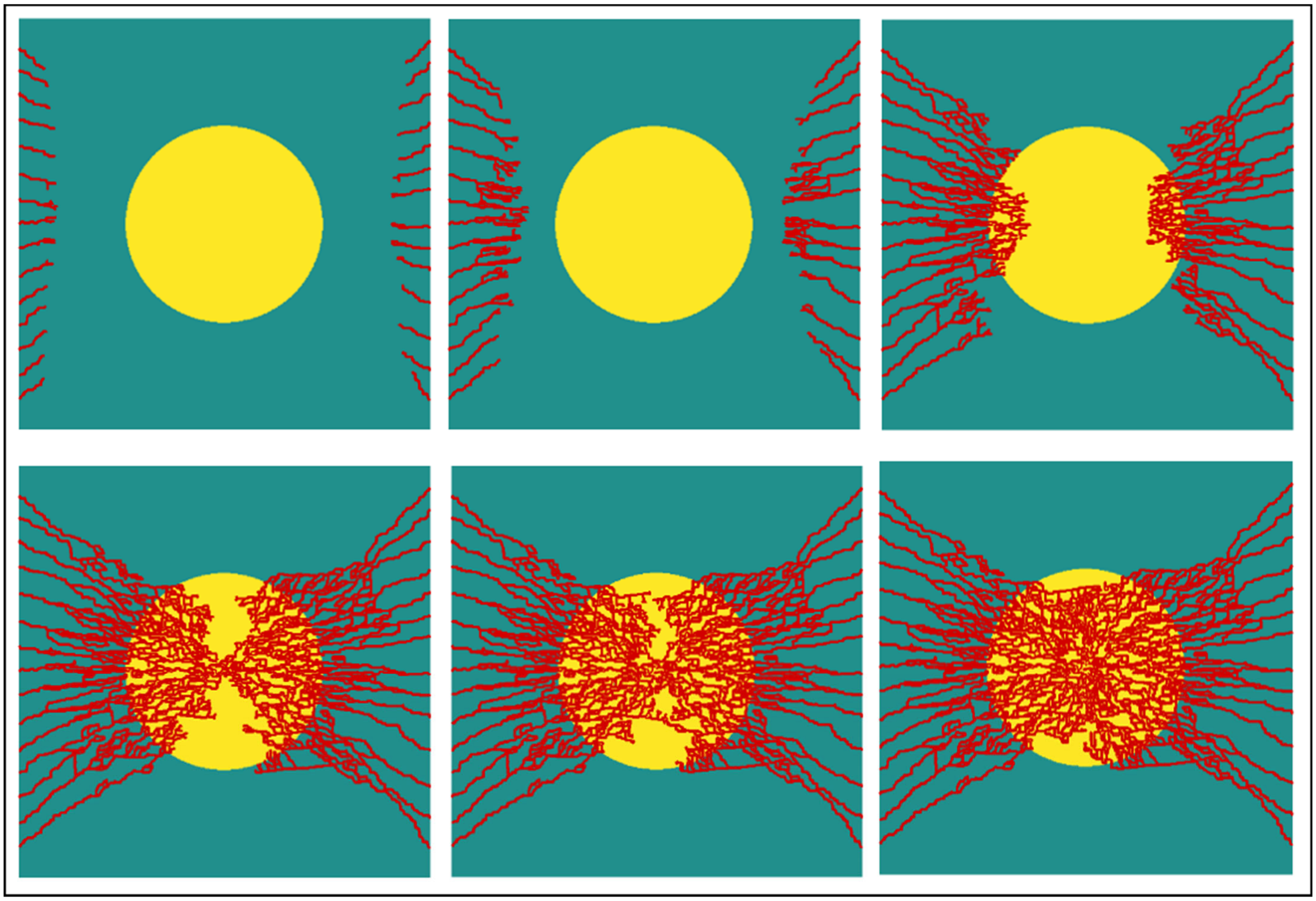

Figure S1. Results of discrete mathematical modeling of tumor-induced angiogenesis for six different stages of capillary networks. The initial sprouts on both vertical sides of the computational domain move toward the solid tumor with a 2.4 cm diameter.

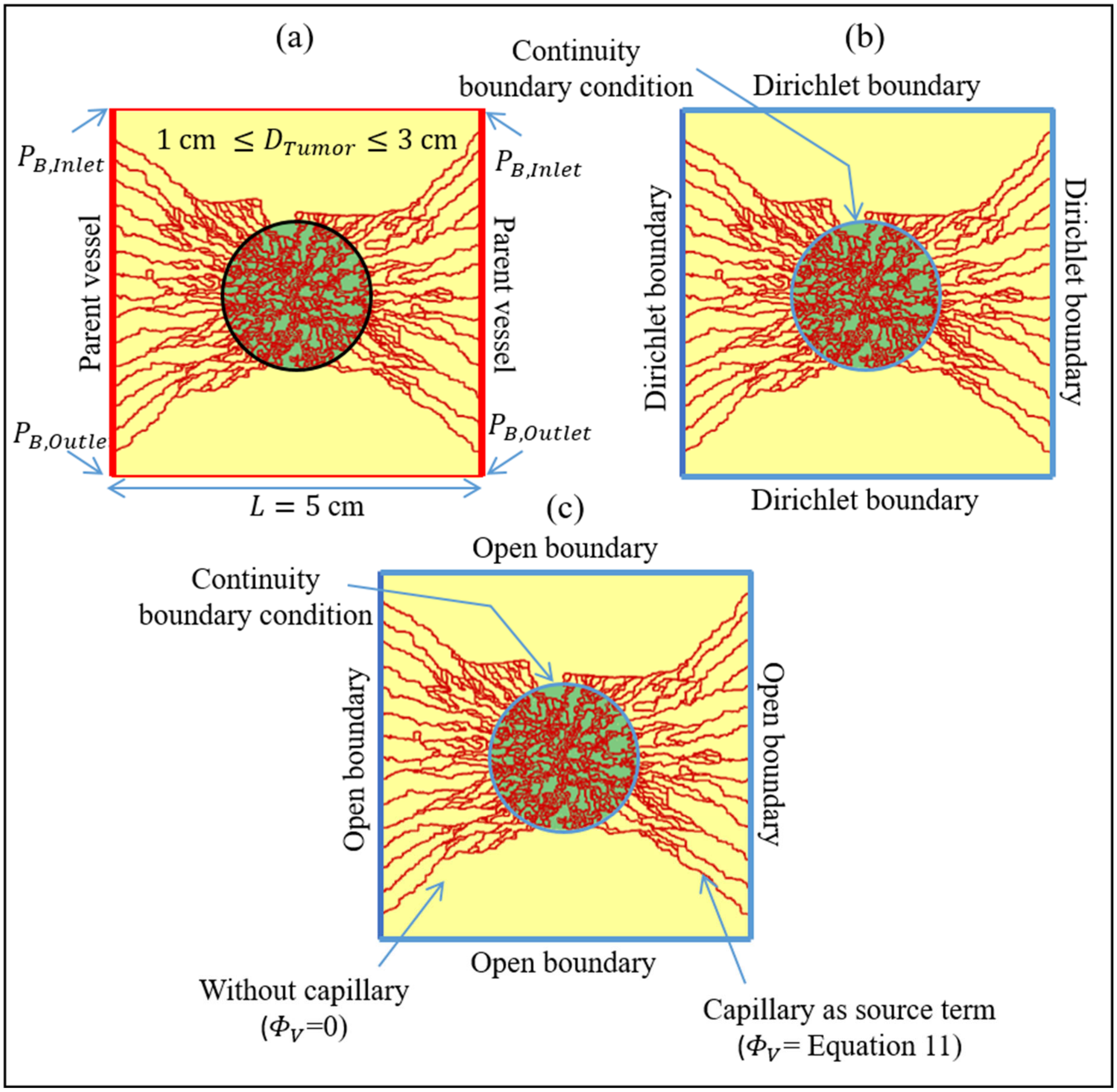

Figure S2. Schematic of the computational domain and implemented boundary conditions. **(a)** The domain consists of a solid tumor with a diameter of D_{Tumor}, surrounding healthy tissue, and tumor-associated vasculatures. Boundary conditions used in the intravascular blood flow simulation are also highlighted. **(b)** Boundary conditions that are used in interstitial fluid flow modeling. **(c)** Boundary conditions used in spatiotemporal modeling of 18F-FDG. The blue arrows indicate the position of different boundary conditions used in each stage of computational simulations.

References

26. Moradi Kashkooli, F.; Abazari, M.A.; Soltani, M.; Akbarpour Ghazani, M.; Rahmim, A. A Spatiotemporal Multi-Scale Computational Model for FDG PET Imaging at Different Stages of Tumor Growth and Angiogenesis. *Sci. Rep.* **2022**. DOI: 10.1038/s41598-022-13345-4.
31. Soltani, M.; Chen, P. Numerical modeling of fluid flow in solid tumors. *PLoS ONE* **2011**, *6*, e20344. https://doi.org/10.1371/journal.pone.0020344.
